# A computational linguistics motivated mapping of ICPC-2 PLUS to SNOMED CT

**DOI:** 10.1186/1472-6947-8-S1-S5

**Published:** 2008-10-27

**Authors:** Yefeng Wang, Jon Patrick, Graeme Miller, Julie O'Hallaran

**Affiliations:** 1 School of Information Technologies, University of Sydney, Sydney, Australia; 2 Family Medicine Research Centre, University of Sydney, Sydney, Australia

## Abstract

**Background:**

A great challenge in sharing data across information systems in general practice is the lack of interoperability between different terminologies or coding schema used in the information systems. Mapping of medical vocabularies to a standardised terminology is needed to solve data interoperability problems.

**Methods:**

We present a system to automatically map an interface terminology ICPC-2 PLUS to SNOMED CT. Three steps of mapping are proposed in this system. The UMLS metathesaurus mapping utilises explicit relationships between ICPC-2 PLUS and SNOMED CT terms in the UMLS library to perform the first stage of the mapping. Computational linguistic mapping uses natural language processing techniques and lexical similarities for the second stage of mapping between terminologies. Finally, the post-coordination mapping allows one ICPC-2 PLUS term to be mapped into an aggregation of two or more SNOMED CT terms.

**Results:**

A total 5,971 of all 7,410 ICPC-2 terms (80.58%) were mapped to SNOMED CT using the three stages but with different levels of accuracy. UMLS mapping achieved the mapping of 53.0% ICPC2 PLUS terms to SNOMED CT with the precision rate of 96.46% and overall recall rate of 44.89%. Lexical mapping increased the result to 60.31% and post-coordination mapping gave an increase of 20.27% in mapped terms. A manual review of a part of the mapping shows that the precision of lexical mappings is around 90%. The accuracy of post-coordination has not been evaluated yet. Unmapped terms and mismatched terms are due to the differences in the structures between ICPC-2 PLUS and SNOMED CT. Terms contained in ICPC-2 PLUS but not in SNOMED CT caused a large proportion of the failures in the mappings.

**Conclusion:**

Mapping terminologies to a standard vocabulary is a way to facilitate consistent medical data exchange and achieve system interoperability and data standardisation. Broad scale mapping cannot be achieved by any single method and methods based on computational linguistics can be very useful for the task. Automating as much as is possible of this process turns the searching and mapping task into a validation task, which can effectively reduce the cost and increase the efficiency and accuracy of this task over manual methods.

## Background

Effective information retrieval across information systems in health care is limited by the lack of semantic interoperability between terminologies used by sectors in the health system. The use of multiple terminologies and ad hoc modifications to standard schemes prevent users from cross searching multiple repositories, cross-sectoral resources and interdisciplinary material. To achieve interoperability and enable exchanging and sharing of data across organisations, the need for improved matching between non-standard terms and a standard medical terminology becomes more important [1,2].

The support of interoperability requires a standard terminology [3] and SNOMED CT is a comprehensive common terminology available in the clinical domain. The use of SNOMED CT to create standardised detailed clinical information will enable more accurate documentation of patient data and improve health quality. The Australian government is proposing to adopt SNOMED CT for describing certain aspects of clinical encounters, coding clinical records, and as a standard terminology in Australian health organisations. This decision creates the need to map existing interface terminologies to the SNOMED CT reference terminology [4]. To complete this task in a reasonable amount of time and improve accuracy, some computational methods of matching concepts between terminologies are needed to assist humans to complete the task.

The process of terminology mapping refers to the identification of identical concepts or relationships between different terminologies. It is an important step to achieving knowledge sharing. Imel and Campbell [5] provide a strong motivation to map medical terminologies, and they assert that the mapping will become increasingly automated leading to increased efficiency and effectiveness. However, the nature of this task makes it very difficult to automate, because heterogeneous terminologies may reflect both fundamentally and subtly different conceptualisations of domains by the authors of these terminologies.

The extensive research done in terminology mapping has had the goal of developing effective automated methodologies for mapping [6-15]. The main approaches include lexical matching, concept matching and structural matching. The earliest approaches were straightforward lexical mapping between terms. The matching is defined on exact string equivalence. Sherertz et al [6] use filters and rules to perform exact lexical matching and they map 834 UCSF (University of Southern California at San Francisco) disease descriptions to Medical Subject Headings (MeSH) terms. They reported 47.8% of the UCSF disease descriptions were mapped to MeSH terms.

Evans et al. [7] and Cimino et al. [8] both used a frame based approach to mapping terms between two vocabularies. A frame is a self-contained unit of knowledge representation that contains a term and its attributes. The idea is to map terms using the attribute and relationship information contained in the frame, such as semantic relationships.

A number of linguists have attempted to make use of linguistic information such as lexical similarity and semantic similarities [9-11]. The National Library of Medicine started the UMLS project in 1986. The SPECIALIST lexicon in UMLS [12] contains a rich set of biomedical terms. Each term contains a base form, abbreviation, and spelling variations. The MetaMap Program [13], created by Aronson et al. utilises the SPECIALIST lexicon to generate lexical variants for concepts in UMLS and can map biomedical text to the UMLS Metathesaurus.

The UMLS has also been used as a resource to integrate between medical vocabularies. Fung and Bodenreider [14] derived an algorithm to find candidate mappings between any two terminologies inside the UMLS making use of synonymy, explicit mapping relations and hierarchical relationships.

Other approaches have been developed recently using structural information to map between terminologies. Mork and Bernstein [15] modified a genetic terminology mapping algorithm for mapping human anatomy, using lexical similarities and structural similarities. However, medical terminologies are different from general terminologies, and they are organised on different axes. Hence, the structural mapping is only moderately effective. Moreover, the medical terminology contains hundreds of thousands of concepts, so searching through all concepts many times is time costly.

To deal with the content completeness problem in terminology mapping, post-coordination has been used to map pre-coordinated terms to compositions of two or more concepts to achieve terminology coverage. Elkin and Brown [16] developed a technique for discovering and formalising the implicit semantic relationships between the SNOMED Reference-Terminology (SNOMED-RT) and the International Classification of Disease Version 9 Clinical Modification (ICD9-CM). Julie Green and her colleagues evaluated an existing model for structured recording of heart murmur findings [17]. They use the *Interprets *and *Has interpretation *concepts in SNOMED CT with a grouping mechanism for roles to represent murmur characteristics and attribute values.

In this paper, we propose an algorithm that automatically maps the International Classification of Primary Care Version 2 (ICPC-2 PLUS) [18], the interface terminology developed in Australia into SNOMED CT [19]. In the process of mapping, we utilised three different mapping strategies to match terms lexically and to perform term decomposition. This mapping process is semi-automatic because it requires humans to verify the results at the completion of the automatic mapping task, but it transforms the time consuming searching and mapping task into an easier selection and validation task. We also have evaluated our mapping algorithm.

## Methods

### Overview of the terminologies

ICPC-2 PLUS is an interface terminology based on the International Classification of Primary Care Version 2 (ICPC-2). It was developed and maintained by The Family Medicine Research Centre (FMRC) of the University of Sydney. The ICPC-2 is a classification designed for general practice and primary care divided into 17 sections such as Musculoskeletal, Neurological, Eye, Blood, etc. The ICPC-2 PLUS is an extension to the ICPC-2 and a version used in Australia. It provides a useable coding system for symptoms, diagnoses, past health problems and the processes of care for use in age-sex disease registers, morbidity registers and full electronic health records in primary care. ICPC-2 PLUS currently contains only 7,410 terms that are commonly used in Australian general practice. It is installed in various software packages and used by approximately 1,500 GPs in electronic health record systems throughout the country.

The Systematized Nomenclature of Medicine Clinical Terminology (SNOMED CT) is developed and maintained by the College of American Pathologists. It is a comprehensive, controlled reference terminology for use in the clinical domain. The basic elements of SNOMED CT are concepts, descriptions, relationships and hierarchies. SNOMED CT contains more than 360,000 concepts, about 1 million descriptions and 1.4 million relationships. Each concept has at least three descriptions including one preferred term, one fully specified name and one or more synonyms. The synonyms provide rich information about the spelling and phraseology variations of a concept, and naming variants used in different countries. The concepts are connected by complex relationship networks that provide generalisation, specialisation and attribute relationships, for example, "focal pneumonia" is a specialisation of "pneumonia". Each concept in SNOMED CT is classified into one of the 18 top categories, including clinical finding, aetiologies, procedures, body part, substances, qualifiers etc.

### The three applied methods

#### The UMLS metathesaurus mapping

The Unified Medical Language System (UMLS) of the National Library of Medicine [22] is a knowledge source that provides the mapping between different terminologies. This is done by incorporating different medical terminologies into a Metathesaurus organized on the basis of a concept. The UMLS Metathesaurus contains information about over one million concepts, 2.8 million distinct strings from over 100 source terminologies and classifications. The 2005AB version of the UMLS contains ICPC-2 PLUS 2000 Version and SNOMED CT terminology, which are the terminologies we needed for our mapping task.

The most direct mapping method used was to utilise the link provided by the UMLS between the two terminologies. The UMLS is organised by concepts, and one of its primary purposes is to connect different names for the same concept from many different vocabularies. Similar terms in different vocabularies are implicitly connected by a unique concept identifier. The idea of our approach is to find the terms in these two terminologies that share a common concept unique identifier (CUI) in UMLS. Every term in the UMLS is represented in a concept structure. The concept structure contains concept identifiers, concept names, their language, and vocabulary source. This information is organised in the *Concept Names and Sources File*. We make use of the common CUI in this file to map terms.

The UMLS mapping requires the latest UMLS Metathesaurus version to achieve the best performance, since the content of the UMLS, and its source vocabularies, are refined and updated regularly. Current experiments were conducted on the 2005AB version which contained a version of ICPC-2 PLUS from 2000, and a version of SNOMED CT from 2002. The version of ICPC-2 PLUS in UMLS accounts for only 87% of the terms currently available in the terminology. ICPC-2 PLUS has since been updated in the UMLS to the most current version, and we therefore expect a larger number of mappings will be discovered when we use the latest version of UMLS.

### String-based mapping

An obvious way to identify mappings between terms is to compare the strings for concept names. The principle behind string-based mapping is that most terminologies have identical or very similar lexical items in their vocabularies for describing the same concepts, as the natural languages underlying the vocabularies are the same. Four string based mapping techniques were used.

#### (i) Normalised Term Matching

Before comparing the string, the terms from both terminologies are normalised using natural language processing techniques. Firstly, words within parentheses are removed. This process removed the suffix attributes in SNOMED CT concepts, for example, "Channel catfish virus disease (disorder)" and "Lump(s);behind ears". Then the terms are tokenised into atomic forms and converted into lowercase. Function words such as "a", "the", "of", "NOS" etc. and punctuation are removed from multi-word expressions. A morphological process is performed on the remaining terms to remove the inflections. Then some common lexical variations of the terms are generated, for example, "haemocyte" to "hemocyte" using the Specialist Lexicon [20] in UMLS. Finally, the remaining words are sorted into alphabetical order. Then the normalised terms are matched using exact string matching method. An example shows the SNOMED CT concept "235856003 Disease of liver (disorder)" is normalized to "disease liver" and can be mapped to ICPC-2 PLUS term "D97002 Disease;liver".

#### (ii) Expanded Term Matching

The Expanded Term Matching process aims to expand the abbreviation of any term to its full form. If the term is not matched in the normalised term matching, the expanded term matching will be performed. There are two kinds of abbreviations found in ICPC 2-PLUS terms. The first is acronyms such as "IUCD" which stands for "Intra-Uterine Contraceptive Device" and the other is abbreviations due to space limitations e.g. "musculo" for "musculoskeletal". These abbreviations cause mismatches in the string matching process, therefore abbreviations are expanded to their full forms. In the first case, a list of acronym to full form mapping is created using the abbreviation list in ICPC 2-PLUS user's guide [18]. In the second case, we adapt the information in the natural language description of the term held in ICPC 2-PLUS to expand the abbreviations. The full form terms are then mapped using the string matching method.

#### (iii) Substring Term Matching

To increase the matching coverage, substring matching is also performed. The pairs of the terms are matched if the normalized and expanded ICPC 2-PLUS term is a substring of the SNOMED CT term. This allows a specific term to map to a general term, for example, the term "chronic pain" is a substring of "chronic back pain". As the source term and target term are similar, but not exact it is possible to produce a large number of invalid matches. The matching process returns the matching candidates ranked using the total proportion of words that are common between the source term and target term. The more words the source and target terms have in common, the higher their rank in the list of candidates.

#### (iv) WordNet Lexicon Matching

This matching approach uses thesauri to explore the semantic variation and meaning of the word constituents. The WordNet synsets [21] were used to provide semantic and syntactic information about the term. The WordNet synset contains a list of synonymous terms for a word constituent. This allows the mapping of "heart disease" into "cardiac disease" because "heart" and "cardiac" are synonyms. WordNet also provides the derivationally related terms for a given word which can be used for searching. For example, the word "fever" is linked to its related adjectives "feverish" and "feverous". Table 1 shows some examples of string-based mapping.

**Table 1:** Examples of string based mapping

**Matching Method**	**ICPC-2 PLUS Term**	**SNOMED CT Term**
Normalised String Matching	L81030 Haemarthrosis;ankle	202415003 Hemarthrosis of the ankle (disorder)
Expanded String Matching	P23008 Disorder;opposit	18941000 oppositional defiant disorder (adolescent)
Substring Term Matching	A85003 Drug Reaction	62014003 Adverse reaction to drug (disorder)
WordNet Lexicon Matching	D21005 Feeling (of);choking	373909009 Choking sensation (finding)

### Post-coordination mapping

The Post-coordination Mapping process aims to map a pre-coordinated ICPC-2 PLUS term to compositions of two or more SNOMED CT concepts, which would thereby constitute a post-coordination in SNOMED CT. This algorithm consists of three steps. Firstly, we break the ICPC-2 PLUS term into atomic terms. This step includes term normalisation, term expansion and separating the words in the text. Then we map each atomic term to the SNOMED CT atomic concepts. The atomic term mapping is based on the longest string match, for example the term "Test;blood;ear" will be broken into three atomic terms "test", "blood" and "ear". The term "Blood test" is then mapped to "Test blood (procedure)" in SNOMED CT rather than mapped to the terms "Test" and "Blood" separately. Finally, we find the relationship between the SNOMED CT concepts by matching the relationship patterns [22] we discovered in SNOMED CT relationships. We aim to map two kinds of post- coordination in SNOMED CT, the Qualification and Combination. Table 2 shows some examples of post-coordination mapping.

**Table 2:** Examples of post-coordination mapping

**Source Term**	**Post-coordinated Concept**
Pain;mouth	22253000 pain (clinical finding) + 21082005 entire mouth region (body structure): relationship type = 363698007 finding site (attribute)
Referral;radiologist	3457005 patient referral (procedure) + 66862007 radiologist (occupation): relationship type = 370131001 recipient category (attribute)
Abuse;verbal ;relative	125677006 Relative (person) + 225825002 Victim of verbal abuse (clinical finding): relationship type = indeterminate
Dislocation;knee;simple	13673007 Simple (qualifier value) + 129156001 Traumatic dislocation of knee joint (clinical finding): relationship type = 246100006 onset (attribute)

### Mapping evaluation

The mapping results were evaluated by two experts (authors GM & JO'H) from the Family Medicine Research Centre at the University of Sydney. They are responsible for developing and refining the ICPC-2 PLUS vocabulary. All mapping candidates generated by our algorithms were exported into a spreadsheet. The experts used the CliniClue SNOMED CT browser to verify the mappings. All the matches were selected on a one to one map of "best-fit". The "best-fit" means the most preferable and suitable match among all ranked candidates. All context dependent concepts in SNOMED CT were excluded as well as legacy concepts. Some matches are of questionable validity due to inappropriate ICPC-2 PLUS mappings to UMLS concepts, and some mappings are reasonable lexical or concept matches but they are category mismatches. Only the "best-fit" matching candidates are considered as the correct matches, the remainder of the matches are considered to be incorrect mappings.

## Results

### UMLS mapping results

There are 13,383 records in the Concept Names and Sources File of ICPC-2 PLUS. It includes the active terms, inactive terms, synonyms, duplicates and language variations (Table 3). By eliminating the synonyms, duplicates, and language variations, 6,502 terms currently have active status, which is 87.75% of the ICPC-2 PLUS vocabulary. These terms are mapped to 6,141 unique Concept Unique Identifiers (CUI) in UMLS.

**Table 3:** Results of mapping using UMLS Metathesaurus


ICPC-2 PLUS entries in UMLS	13,383
Active ICPC 2-PLUS terms in UMLS	6,502
Number of mapped CUI	6,141
Number of ICPC-SNOMED CT mapping candidates	6,557
Number of best-fit mapping	3,326
ICPC 2-PLUS term mapped to SNOMED	3,448 (53.0%)
Correct ICPC 2-PLUS mappings	3,326 (96.5%)
Average number of mapping per term	1.9

The UMLS mapping algorithm mapped a total of 3,448 ICPC 2-PLUS terms (53.0% of active terms) to SNOMED CT concepts through 6,557 Common Unique Identifiers in UMLS, which is an average of 1.9 mappings per ICPC-2 PLUS term. In the evaluation, only one-to-one best fit matching was considered a correct mapping. Hence, among the 6,557 mapping candidates, 3,326 (50.72% of the total candidates) one-to-one mappings were manually evaluated as correct mapping candidates resulting in a precision rate of 96.46% and recall rate of 44.89%.

### String-based mapping result

These experiments were run in a cascaded manner. The normalised term mapping was performed first, then expanded matching on the rest of the unmatched terms, and similarly for WordNet Mapping and Substring mapping. A total of 3,266 ICPC-2 PLUS terms (44.5% of all ICPC terms) were mapped to SNOMED CT terms using normalized string matching (Table 4). This matching method generated a total 3,565 mapping candidates, on average, 1.2 matches per matched terms. The majority of matched terms were single word terms and multi-word expressions. Some terms with different spelling variations were also mapped. The Expanded String Matching further mapped 304 terms. It effectively increased the number of mappings in chapter L (Musculoskeletal) of ICPC-2 PLUS, because most of the terms in this chapter were compressed to a short form, however, the average mappings per term increased to 1.33. WordNet Lexicon Matching is not very effective and only gave a 1% increase in mapping coverage. Most of the Substring Matching results were one to many, and the average number of matches per term increased to 24.88.

**Table 4:** String-based mapping results

**Matching Method**	**Overall Matched**	**Overall %age**	**Newly Mapped**	**Correct Matching**	**Accuracy**
Normalized String Matching	3,266	44.08%	3,266 (44.08%)	3,031	92.80%
Expanded String Matching	3,570	48.18%	304 (4.10%)	287	94.41%
WordNet Matching	3,662	49.42%	92 (1.24%)	80	86.96%
Total Substring Matching	4,471	60.34%	809 (10.88%)	-	-

Overall, normalised matching mapped 3,266 (44.08%) terms, Expanded String Matching further mapped 304 (4.10%) terms, WordNet Matching give another 92 (1.24%) newly mapped terms, and Substring Matching increased by 809 (10.88%) the matched terms. The combined string matching methods gives an overall of 4,471 (60.30%) mapped ICPC 2-PLUS terms.

The string matching results were evaluated by the same experts. Similarly to the UMLS evaluation, only the "best-fit" matches were considered as correct matches. 3,031(92.8%) of the 3,266 normalised matching terms had at least one correct mapping candidate. Among 304 Expanded matching terms, 287 (94.41%) terms were correct mappings. 80 (86.96%) out of 92 WordNet mappings were correct mappings. The results of substring matching were not evaluated, as the number of matching candidates increased to 24.88 per term on average. However, by observation, 80% of the substring matching had at least one correct matching. Overall 3,662 terms in string matching results were evaluated, 3,398 terms were a correct matching, which results in a precision of 92.79%, and recall rate of 45.86%.

Several mismatched terms were due to coordination of the terms, or the term was connected with conjunctions, slashes etc. such as the term "Splint/immobilise; nerve". Category mismatches occur when the source term and target term have strong lexical similarity but belong to different categories. For example the ICPC 2-PLUS term "A59007: Pain management" is mapped to SNOMED CT concept "394882004 pain management (speciality)", whereas it should be matched to "278414003 pain management (procedure)".

### Post-coordination mapping result

Post-coordination mapping was performed on the terms remaining after the terms that had been mapped in the previous mapping algorithms were excluded. The remaining set consisted of 3,840 terms (Table 5). These terms do not have any string matches in SNOMED CT terminology nor can they be expressed using one single SNOMED CT concept.

**Table 5:** Post-coordination mapping results

**Post-coordination Type**	**Number of Mappings**	**Percentage**
Qualification	343	4.63%
Combination	902	12.17%
Indeterminate	255	3.44%
Total	1500	20.24%

There are three types of mappings that can match ICPC 2-PLUS terms to SNOMED CT post-coordinations: Qualification, Combination, and Indeterminate. *Qualification *is a match with a post-coordinated SNOMED CT concept that has at least one qualifier value concept. *Combination *is a mapping that produces a set of SNOMED CT concepts which does not include any qualifier value, and where the relationships between the concepts can be identified. *Indeterminate *mappings identify post-coordinations that match a set of SNOMED CT concepts, but the relationship between the concepts could not be determined from the SNOMED CT data distribution. Overall there were 20.24% terms mapped using post-coordination.

## Discussion

As the number of medical terminologies increases, greater demands for the need for terminology integration arise. As a result, the demand for rapid and effective computer-assisted terminology mapping has arisen. Computerised mapping systems could significantly reduce human effort, especially for mapping between large terminologies. While the system is able to automatically generate potential matches, human coders still need to validate the results from a list of matching candidates. However, automation of this process significantly reduces the human effort because it transforms the time consuming searching and matching tasks into selection and validation tasks.

The mapping provided by the UMLS Metathesaurus can be considered as a golden standard. By observation, a large percentage of the mappings provided by the UMLS Metathesaurus are lexical mappings. However, the mapping still produces on average 1.9 mappings per term and some of the mappings are still ambiguous. Using the preferred term in SNOMED CT descriptions as the one to one mapping reduces the accuracy of mappings because the SNOMED CT terminology is developed in America and the preferred terms are in American English. As the preferred term and synonyms for the same concepts are used differently in Australia, manual validation of the mappings is still need.

On evaluation, the normalised string matching and expanded string matching were accurate and useful for about 50% of the ICPC-2 PLUS terms. The substring matching had broader coverage, but resulted in a large number of mapping candidates. Upon inspection, a lot of substring mappings were imprecise. Nevertheless, roughly 10% of the mappings were still accurate. One possibility for reducing the superfluous mapping candidates in string-based mapping could be to use the semantic information and categorical information in the SNOMED CT hierarchy.

Initially, we expected that the structural information of these two terminologies could have provided some useful clues for the matching, however these two terminologies are organised differently. The ICPC-2 PLUS has a biaxial structure and the sections are organised on the body system and social problems, whereas SNOMED CT is based on 18 key classes. The different organisation of these two terminologies makes it is difficult to utilize the structural information.

The use of synonyms in WordNet is not very useful. By looking at the results, we found that the synonym concepts in SNOMED CT descriptions are able to capture most of the synonyms in WordNet. The results of the WordNet mapping is not as effective as the work done by Mougin [23] because the matching criteria we used is restricted to produce less ambiguity in matching candidates. There is a trade off between the coverage of potential mapping produced by the algorithm and the accuracy of mapping.

The results of post-coordination mapping have not yet been evaluated. Nevertheless, the system has demonstrated its ability for automated term decomposition using a combination of string-based mapping techniques. One important phenomenon in post-coordination is the identification of relationships between the mapped terms. This may require description logic generation and more detailed semantic analysis to make sure the matching of two concepts makes sense. We believe that the post-coordination mapping is a way to solve the content completeness problem among different terminologies.

## Conclusion

In conclusion, we have mapped about 80.58% of ICPC-2 PLUS terms to SNOMED CT concepts with differing levels of accuracy via three automated mapping approaches. This research has demonstrated that automated mapping based on computational linguistic principles can perform different levels of terminology mapping. The results have shown that some of the mapping methods produce very reliable mapping, while some methods yield broader coverage but less convincing selections. The mapping results provide an opportunity to analyse the differences in these two different terminologies. Further refinement of the mapping methods could be done to reduce superfluous and incorrect mapping using structural and categorical information, for example, the elimination of synonym ambiguity. Also, more sophisticated post-coordination mapping could be developed in order to provide more reliable mapping.

## Competing interests

Authors Miller and O'Hallaran are employed to manage and maintain ICPC-2 PLUS.

## Authors' contributions

Wang and Patrick were responsible for the computational solutions and processing. Miller and O'Hallaran were responsible for supplying the ICPC 2-PLUS expertise and completing the manual checking of all computed matches.
